# Quantitative Plasma Proteomics to Identify Candidate Biomarkers of Relapse in Pediatric/Adolescent Hodgkin Lymphoma

**DOI:** 10.3390/ijms23179911

**Published:** 2022-08-31

**Authors:** Ombretta Repetto, Laura Caggiari, Mariangela De Zorzi, Caterina Elia, Lara Mussolin, Salvatore Buffardi, Marta Pillon, Paola Muggeo, Tommaso Casini, Agostino Steffan, Christine Mauz-Körholz, Maurizio Mascarin, Valli De Re

**Affiliations:** 1 Facility of Bio-Proteomics, Immunopathology and Cancer Biomarkers, CRO Aviano, National Cancer Institute, IRCCS, 33081 Aviano, Italy; 2 AYA Oncology and Pediatric Radiotherapy Unit, CRO Aviano, National Cancer Institute, IRCCS, 33081 Aviano, Italy; 3 Istituto di Ricerca Pediatrica Città della Speranza, 35127 Padova, Italy; 4 Department of Pediatric Hemato-Oncology, Santobono-Pausilipon Children’s Hospital, 80123 Napoli, Italy; 5 Pediatric Hematology, Oncology and Stem Cell Transplant Division, Padova University Hospital, 35128 Padova, Italy; 6 Department of Pediatric Hemato-Oncology, University Hospital of Policlinico, 70124 Bari, Italy; 7 Division of Pediatric Oncology/Hematology, Meyer University Children’s Hospital, Via Gaetano Pieraccini 24, 50139 Florence, Italy; 8 Department of Pediatric Oncology, Zentrum für Kinderheilkunde der Justus-Liebig-Universität Gießen, UKGM Standort Gießen, 35392 Giessen, Germany

**Keywords:** biomarker, label-free quantification, mass spectrometry, pediatric Hodgkin Lymphoma, proteomics, relapse, C4b-binding protein α chain, clusterin

## Abstract

Classical pediatric Hodgkin Lymphoma (HL) is a rare malignancy. Therapeutic regimens for its management may be optimized by establishing treatment response early on. The aim of this study was to identify plasma protein biomarkers enabling the prediction of relapse in pediatric/adolescent HL patients treated under the pediatric EuroNet-PHL-C2 trial. We used untargeted liquid chromatography-tandem mass spectrometry (LC-MS/MS)-based proteomics at the time of diagnosis—before any therapy—as semiquantitative method to profile plasma proteins specifically associated with relapse in 42 children with nodular sclerosing HL. In both the exploratory and the validation cohorts, six proteins (apolipoprotein E, C4b-binding protein α chain, clusterin, fibrinogen γ chain, prothrombin, and vitronectin) were more abundant in the plasma of patients whose HL relapsed (|fold change| ≥ 1.2, *p* < 0.05, Student’s *t*-test). Predicting protein function with the Gene Ontology classification model, the proteins were included in four biological processes (*p* < 0.01). Using immunoblotting and Luminex assays, we validated two of these candidate biomarkers—C4b-binding protein α chain and clusterin—linked to innate immune response function (GO:0045087). This study identified C4b-binding protein α chain and clusterin as candidate early plasma biomarkers of HL relapse, and important for the purpose of shedding light on the molecular scenario associated with immune response in patients treated under the EuroNet-PHL-C2 trial.

## 1. Introduction

Classical Hodgkin lymphoma (HL) is the most common cancer in childhood, that can usually be treated successfully (the overall 5-year survival rate is >95%) [1]. The Hodgkin and Reed-Sternberg tumor cells involved are surrounded by a dense and complex microenvironment that supports their survival and proliferation [2,3].

Current HL therapies achieve high cure rates, but also carry a risk of therapy-related toxicities [4,5,6], especially in cases of relapsing/refractory disease, which can be cured with high-dose therapy, as reviewed in Daw et al. [7]. In the clinical management of HL, one of the main goals is to reduce the long-term toxicities of radiotherapy and chemotherapy by minimizing the treatments’ intensity based on the risk category of the disease. The recent findings of the European EuroNet-PHL-C1 trial suggest that 40% of Group 2 and Group 3 patients—i.e., those with an adequate response after two cycles of OEPA chemotherapy, consolidated with COPP or COPDAC—could safely be spared radiotherapy [8].

There is also great interest in identifying biomarkers capable of serving as predictors of tumor progression and relapse, which may justify treatment intensification. Decreasing levels of albumin and hemoglobin, leukocytosis and lymphocytopenia had been proposed as predictors of a worse outcome in adults [9]. A low erythrocyte sedimentation rate—ESR ≤ 20 mm/h—was identified as a significant predictor of event-free survival in stage IA or IIA, nonbulky disease HL [10] in the Children’s Oncology Group study AHOD0431. In the current EuroNet-PHL-C2 trial ESR ≥ 30 mm/1st h has been chosen for stratifying low risk patients into a higher treatment level.

The prognostic significance of the blood proteome has not been investigated extensively in pediatric HL. Some studies using targeted single-protein assays (e.g., plasma heparanase [11] and serum VEGF [12]), enzyme-linked immunosorbent assays (ELISA) and, more recently, Luminex multiplex assays (e.g., TNF superfamily member 10) [13] found that blood proteins may be helpful in HL prognostics, but none of them are used in clinical practice today.

In a previous study, we applied a liquid chromatography-tandem mass spectrometry (LC-MS/MS)-based proteomic approach to adolescents treated according to the LH2004 chemo-radiotherapeutic HL protocol. We identified a set of biomarkers, some predictive of HL relapse (e.g., fibrinogen α and γ chains), and others protective against relapse (i.e., α-1-antitrypsin and antithrombin III) [14]. LC-MS/MS is an analytical strategy widely used nowadays in biomedical translational research to find biomarkers in blood [15,16]. In label-free LC-MS/MS approaches, the relative abundance of different proteins is measured from mass spectral peak intensities or by spectral counting [17], and protein expression patterns can be compared across samples. Using this approach has enabled the discovery of numerous potential biomarkers (e.g., [18,19,20]). In the present work, label-free LC-MS/MS was used to identify circulating biomarkers capable of predicting relapse in a case/control study on 42 children with HL treated according to the European Network for Pediatric Hodgkin Lymphoma (EuroNet-PHL) C2 protocol (NCT02684708).

## 2. Results

### 2.1. Clinicopathological Characteristics and Hematological Parameters

Table 1 shows the characteristics of patients in the exploratory and validation cohorts by disease status, after first-line treatment according to EuroNet-PHL-C2 trial (for each cohort: relapsed *n* = 7; non-relapsed *n* = 14). All patients had the NS-HL subtype.

None of the laboratory-tested blood parameters (Plts, WBC, IgG, IgA, IgM, albumin, CRP, ferritin, fibrinogen, Hb, total protein) differed significantly between the relapsed and non-relapsed pediatric/adolescent HL patients in either of the cohorts (*t*-test, *p*-value > 0.05; Table S1).

### 2.2. Plasma Protein Profiling

**Figure 1:**
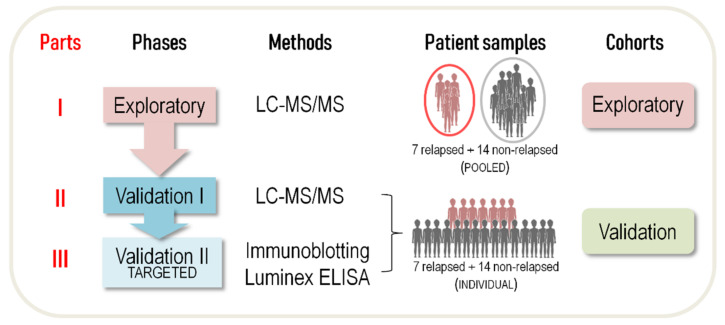
The three parts of our experimental workflow. LC-MS/MS, label-free quantitative liquid chromatography-tandem mass spectrometry; ELISA, enzyme-linked immunosorbent assay.

**Figure 2:**
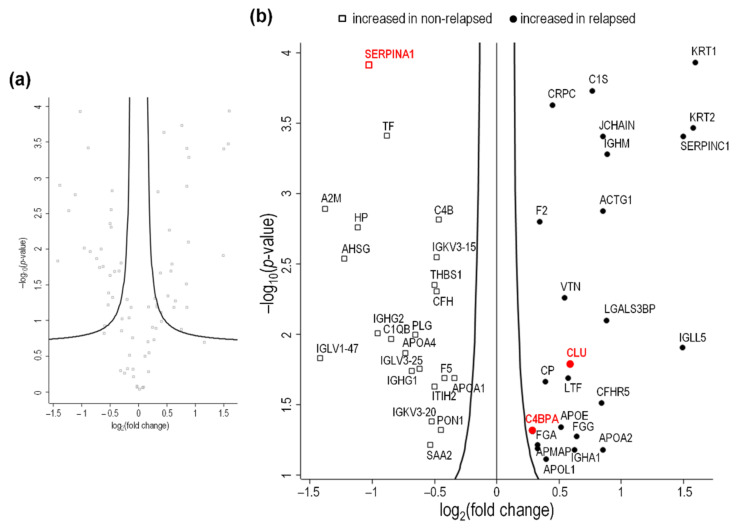
Volcano plot showing proteomic data by HL status (relapsing vs. non-relapsing) in the exploratory cohort. Log2 transformed abundance ratios for each protein are plotted on the *x*-axis. Negative log10 transformed *p*-values are plotted on the *y*-axis. The 107 proteins identified and quantified are shown in grey in (**a**). Among them, there were 24 proteins (circles) significantly more abundant, and 22 (squares) less abundant in patients whose HL relapsed than in non-relapsing patients (**b**). Proteins selected in Part II are evidenced in red.

Our study design is illustrated in Figure 1. In Part I of the study (exploratory phase), depleted plasma samples obtained before any therapy and pooled according to patients’ HL relapse status were analyzed using label-free LC-MS/MS (Figure S1). A total of 107 proteins were quantified and identified (Supplementary File Proteome_data, sheet 1). Quantitative comparisons between the relapsed and non-relapsed groups identified 24 proteins that were significantly more abundant in the plasma of patients who experienced relapse, and 22 proteins that were more abundant in the plasma of non-relapsing HL patients (*t*-test, *p*-value < 0.05) (Figure 2; Table 2).

In Part II of our study (validation phase), label-free LC-MS/MS analysis identified a total of 170 proteins differing in abundance between the relapsed and non-relapsed HL groups in our validation cohort (Supplementary File Proteome_data, sheet 2). Twenty-three of these proteins were more abundant in the relapsed HL group (*p* < 0.05; Table S2). Principal component analysis of the 170 differently-expressed proteins was able to discriminate this group from the non-relapsed group, albeit with a marked biological variability from patient to patient (Figure S2). This result confirmed the power of the protein profile in discriminating patients by relapsed vs. non-relapsed HL status.

The following six proteins were confirmed as being more abundant in relapsed than in non-relapsed HL patients (in alphabetical order): apolipoprotein E (APOE), C4BPA, CLU, fibrinogen γ chain (FGG), prothrombin (F2), and vitronectin (VTN). Among the proteins found less abundant in relapsed than in non-relapsed HL in Part I of our study, lower levels of SERPINA1 was not confirmed in the validation phase. We had previously identified this protein using label-free LC-MS/MS and two-dimensional differential gel electrophoresis [14,21]. Differences in its abundance were assessed using other approaches in Part III of this study.

**Figure 3:**
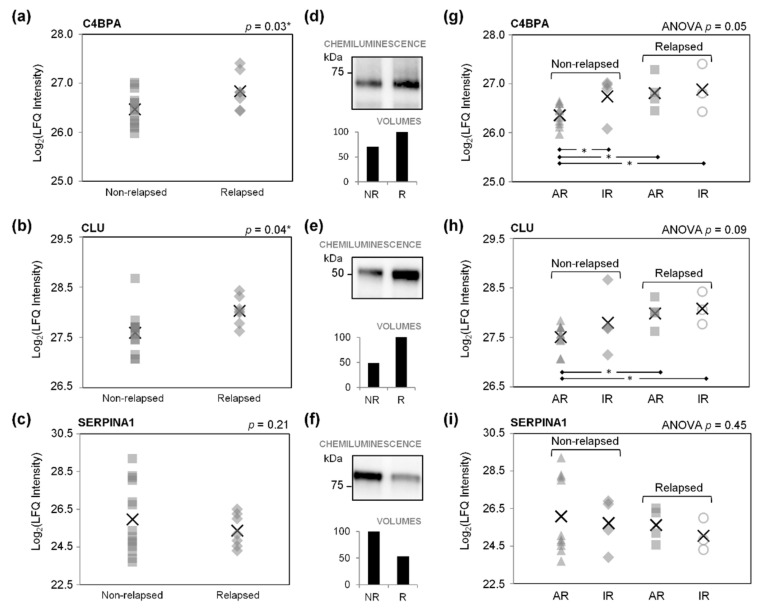
Abundance of C4b-binding protein α chain (C4BPA), clusterin (CLU), and α-1-antitrysin (SERPINA1) in pediatric/adolescent patients with non-relapsed (NR) and relapsed (R) HL (validation phase). Non-relapsed and relapsed patients were divided according to whether or not they had an adequate (AR) or inadequate (IR) response to therapy on early response assessment, as explained in the Materials and Methods. Data are expressed as LFQ values obtained with LC-MS/MS (**a**–**c**,**g**–**i**), or as band intensities from immunoblotting (**d**–**f**). In (**a**–**c**,**g**–**i**), the crosses indicate the mean value of the parameter tested, and the other symbols (squares, rhombuses, triangles, and circles) indicate individual patients. * Significance for *p*-value < 0.05. NR, non-relapsed; R, relapsed.

According to DAVID Bioinformatics Resources, the proteins found more abundant in relapsed HL in both the exploratory and the validation phases of our study share the following four biological processes: “innate immune response” (GO:0045087; including CLU and C4BPA), “fibrinolysis” (GO:0042730; including F2 and FGG), “blood coagulation” (GO:0007596; including F2 and FGG), and “positive regulation of neurofibrillary tangle assembly” (GO:1902998; including CLU and C4BPA) (Supplementary File DAVID_raw data, sheets 1 and 2). We focused on the “innate immune response”, and on C4BPA and CLU as two independent proteins more abundant in relapsed HL (*p* < 0.05; Figure 3a,b) in our cohorts of pediatric/adolescent patients with HL.

**Figure 4:**
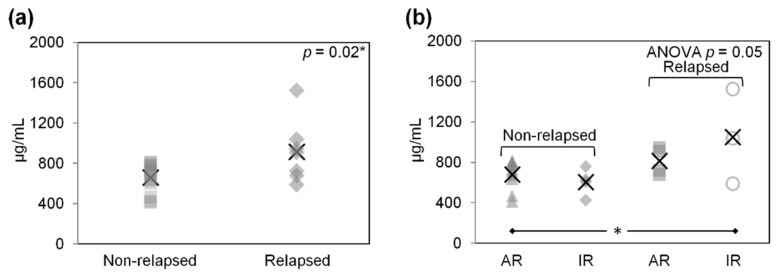
Abundance of clusterin (CLU) (µg/mL) in non-relapsed and relapsed pediatric/adolescent HL patients, measured using Luminex-based assays (validation phase). In (**b**), the non-relapsed and relapsed patients shown in (**a**) are divided according to their adequate (AR) or inadequate (IR) response to therapy on early response assessment, as explained in the Materials and Methods. In (**b**), ANOVA *p*-values are given in the top right-hand corner of the graph. Symbols identify individual patients, and crosses indicate mean values. * significance for *p*-value < 0.05, Student’s *t*-test.

In Part III, differences in the abundance of C4BPA and CLU were examined by immunoblotting plasma proteins from the relapsed and non-relapsed HL groups (pools of 7 and 14 samples, respectively). The higher levels of both C4BPA and CLU in relapsed HL plasma were confirmed (Figure 3d,e). The greater abundance of CLU in relapsed HL plasma was also confirmed using a Luminex-based assay (*p* < 0.05; Table S3; Figure 4a).

Irrespective of the adequacy of their response on ERA, relapsed patients had significantly higher mean C4BPA and CLU levels than non-relapsing patients with an adequate response on ERA (*p* < 0.05; Figure 3g,h). The C4BPA levels varied significantly in the four study groups (one-way ANOVA *t*-test *p* = 0.05; Figure 3g). Luminex-based analysis only confirmed the higher CLU levels in relapsed patients with an inadequate response on ERA as compared with non-relapsed patients whose response was adequate (*p* = 0.05; Figure 4b).

Higher levels of SERPINA1 were found in the plasma of non-relapsed HL patients on immunoblotting (Figure 3f), and also—though the differences were not significant—on LC-MS/MS (*p* = 0.21; Figure 3c), Luminex-based arrays (*p* = 0.27; Table S3; Figure S3a), and ELISA assays (*p* = 0.07; Figure S3c). A slightly higher SERPINA1 content was found in non-relapsed patients, particularly those showing an AR on ERA, by comparison with relapsed patients, irrespective of whether they achieved an AR or IR on ERA (Figure 3i and Figure S3b).

## 3. Discussion

The present study identified six proteins (APOE, C4BPA, CLU, FGG, F2, and VTN) in greater abundance in the plasma obtained at diagnosis from Italian patients with HL who relapsed after first-line treatment. According to DAVID, the plasma was rich in proteins participating in the biological process of “innate immune response”, including C4BPA and CLU. These findings are consistent with those of our previous study, which found an association between immune response and a worse response to treatment in the LH2004 clinical trial [14]. Several mechanisms of immune escape, self-destruction avoidance, and aberrances in the innate immune system have been described for HL reviewed in [22].

Our present study focused on the correlation of C4BPA and CLU with any prognostic impact and immune response in classical HL treated under the EuroNet-PHL-C2 protocol. The higher abundance of C4BPA and CLU found by untargeted, label-free LC-MS/MS in our exploratory and validation cohorts was confirmed by immunoblotting and Luminex-based arrays. We also found an association between lower SERPINA1 levels and relapsing HL, though the difference in median abundance was only significant for the ELISA tests. In our previous studies regarding the LH-2004 clinical trial, we also found a correlation between higher SERPINA1 levels and a better prognosis in HL [14,21].

Our label-free LC-MS/MS-based semiquantitative proteomic analysis succeeded in identifying quantifiable differences in protein levels across patients with and without relapsing HL. This powerful technology has been increasingly used in cancer research and biomarker discovery in recent years [23]. The small size of our cohorts prompted us to adopt a triangular strategy for plasma proteome profiling, while the more attractive idea of using larger cohorts in a rectangular strategy (described in [24]) remains a prospect for further investigations.

The use of label-free LC-MS/MS method is generally considered the choice of method for proteomic discovery studies. As an untargeted shotgun method, the label-free LC-MS method may identify and quantify a large number of plasma proteins with a high specificity, that may include false-positive results in spite of the use of the Bonferroni correction [25]. It suffers from accurate quantitation, thus performing relative quantitation and generating candidates, that need to be validated using targeted approaches. In our study, the three selected proteins (C4BPA, CLU and SERPINA1) were validated by ELISA and Luminex methods, that have the advantages of high sensitivity, but they are limited to antibody availability and may be costly if measuring multiple proteins. We deserve to continue our validation processes by other targeted MS techniques, such as MRM/PRM (reviewed in [25]), able to detect and quantify proteins with precision across samples, in the future in a large cohort of patients.

We identified a total of 107 and 170 plasma proteins, respectively, in our exploratory and validation LC-MS/MS analyses, numbers that are consistent with other recent, label-free, MS-based quantitative proteomic investigations on depleted plasma/serum and cancer (124 in non-small cell lung cancer [19]; 140 in pancreatic cancer [26]; 176 in oropharyngeal cancer [27]). These low numbers of proteins identified may stem from our choice to use immunodepletion to reduce the extremely high dynamic range of plasma: this procedure has the drawback of normalizing the plasma proteome with the risk to under-represent the less abundant non-targeted proteins [28]. This limitation of our experimental design could be overcome by the use of other methods to deplete plasma (such as affinity capture enrichment, described in [29]) or working with undepleted plasma by running multiple fractionation steps, and using different MS scanning methods [30,31] to improve the detection levels.

The differences between the plasma proteins recovered using LC-MS in our exploratory and validation phases may stem from the different sources of samples: the exploratory analysis was performed on pooled plasma, that has the advantage to overcome the high biological variability of plasma, while the validation analysis on individual patients’ plasma, where the high physiological intra-individual differences emerged in protein levels. This may also explain why we only found SERPINA1 more abundant in pooled non-relapsed HL plasma (in the exploratory phase), in agreement with our immunoblotting results and previous work [14]. Our ELISA and Luminex immunoassays on individual patients’ plasma did not confirm these higher levels of SERPINA1 in non-relapsed HL, although they did reveal the presence of a high protein content in some patients. It is worth noting that our validation analyses in Part III of our study were performed on frozen plasma collected before and stored at −80 °C for longer than the plasma used in Parts I and II, and the possibility of different pre-analytical variables negatively affecting plasma protein concentration/stability cannot be excluded.

All the proteins that we found differed in abundance between our groups with relapsing as opposed to non-relapsing HL are secreted in plasma, but also intracellular; and some of them have been found expressed in cancer tissue. CLU expression may occur in malignant lymphoma, for instance, including HL [32]. Further investigations may shed light on the higher or lower levels of the proteins we investigated as biomarkers of tumor relapse, and their possible role(s) in the HL tissue microenvironment. Validation analyses should also continue on the other four proteins found more abundant in the plasma of patients whose HL relapsed.

This study has some other limitations, together with those described above. First, our sample was small and may only partly reflect the real impact of HL on the plasma proteome. Our analyses should be repeated on larger cohorts of patients with a longer follow-up, to allow a stratified analysis even between different risk groups (TL2 versus TL3). In this work, our small case number come from our criteria to select control patients (non-relapsing HL) matching for age, sex, NS histological subtype, and clinicopathological characteristics with the case ones (relapsing), who were few in number. Another limit of our work is linked to the different randomization of patients (COPDAC versus DECOPDAC consolidation cycles) that could affect the risk of relapse. Actually, the final results of the trial are not yet available. Another limitation lies in the marked heterogeneity and complexity of the biological matrix analyzed: blood reflects the physiological status of an individual, so that protein profiles in plasma vary considerably, depending on numerous factors (such as age, sex, hormones, and metabolism). In spite of these factors and its wide dynamic range—plasma contains proteins covering 10-order-of magnitude range of protein concentration—this matrix, considered as “the most difficult-containing sample to characterize” [33], is a still promising biofluid of choice, more reproducible than serum, containing proteins that reflect a variety of human disease and thus promising for studies applied to biomarker discovery.

To date LC-MS-based quantitative proteomics analytical workflow allowed to discover several novel potential cancer biomarkers in serum/plasma for clinical application (reviewed by [15]): for instance, recently, altered protein abundances were found in non-Hodgkin lymphoma, infected or not by human immunodeficiency virus, (i.e., C1Q and B2M) [34]; predicted the outcome of breast cancer patients receiving neodiuvant chemotherapy (APOC3, MBL2, ENG and P4HB) [35] or associated with high risk of ovarian cancer in BRCA1/2 carriers (SPARC and THBS1) [20].

### Possible Role of Differences in the Abundance of Proteins in Relapsed HL

C4BPA is a plasma glycoprotein synthesized in the liver [36] and activated monocytes [37]. It is one of the major inhibitors of the classical and lectin pathways of complement activation [38]. It has various isoforms, and the one called C4BPA is generally more expressed in the case of inflammation, which is known to occur in HL [13]. One of the various functions of C4B-binding proteins is to induce B cell survival and proliferation by linking CD40 [39], a receptor of the tumor necrosis factor superfamily [40] strongly expressed in HRS cells [41]. To the best of our knowledge, the present study is the first to describe circulating C4BPA in HL, and how it varies on the proteomic level between pediatric/adolescent patients with relapsed and non-relapsed HL.

CLU is an 80 kDa heterodimeric glycoprotein ubiquitously and constitutively expressed in several tissues and body fluids [42]. Traditionally described as one of the most prominent chaperones in extracellular and intracellular proteostasis [43], CLU is involved in a plethora of fundamental processes with multiple, and sometimes opposing roles (reviewed in [44]). Many studies have examined secretory CLU (sCLU) as the protein’s concentration increases in various types of cancer (breast [45]; ovarian [46]; prostate [47]), and regulates a whole range of oncogenic signaling pathways (reviewed in [48]). Interestingly, it has been demonstrated that sCLU is a stress-inducible protein in HL with an active role in the immune response to chemotherapy and ionizing radiation [48], and in breast cancer it has been correlated with invasiveness and prognosis [45].

High-throughput functional and in vitro validation studies will be needed to assess these candidates’ potential as biomarkers for predicting relapse in pediatric/adolescent patients with HL.

## 4. Materials and Methods

### 4.1. Treatment Protocol and Research Ethics Statement

This study concerns 42 children and adolescents with HL in Italy enrolled onto the EuroNet-PHL-C2 trial (NCT02684708) from May 2016 to December 2020.

The EuroNet-PHL-C2 is a phase III, multicenter, randomized, controlled trial for all patients with first-line classical HL under 18 years old (or under 25 in Italy, France and UK). All patients were treated with 2 induction OEPA cycles. Intermediate (TL-2, namely stage IA/IB/IIA with ESR ≥ 30 or with bulk, IAE/IBE/IIAE/IIB/IIIA) and advanced stage (TL-3, namely stage IIBE, IIIAE, IIIB, IV) patients were then randomized between 2 or 4 standard COPDAC-28 or intensified DECOPDAC-21 consolidation chemotherapy cycles, respectively, for TL-2 or TL-3 level. The main purpose of the study was to investigate whether: (i) EFS in patients with an adequate response (Deaville scores 1, 2, 3) on early response assessment (ERA), can be improved by using a new strategy of intensified consolidation chemotherapy (DECOPDAC) without radiotherapy; and (ii) in patients with an inadequate response on ERA (Deauville scores 4, 5), the intensified consolidation chemotherapy, with a reduced volume of radiotherapy, only to the residual nodes at the end of chemotherapy, is comparable to standard consolidation chemotherapy plus involved fields radiotherapy to the affected nodes at diagnosis [49,50].

This trial was approved by the CRO Institutional Review Board (decision no. CRO-2016-12; 7 March 2016), and by the ethics committees of the participating institutions in Italy. Written informed consent to the use of patients’ plasma samples in future research was obtained for all patients from their parents or legal guardians.

### 4.2. Patient Selection and Plasma Sampling

Plasma samples from Italian pediatric/adolescent patients with classical HL enrolled onto the EuroNet-PHL-C2 trial were collected at diagnosis, before any treatment. This matched case/control study involved 14 cases of relapsing classical HL of nodular sclerosing (NS) histological subtype, and 28 controls, defined as non-relapsing HL patients. Controls were selected on the basis of a case/control ratio of 1:2, and matched for age, sex, NS histological subtype, and clinicopathological characteristics, i.e., stage, systemic symptoms, negativity for Epstein-Barr virus (EBV), and intermediate (TL2) or advanced (TL3) treatment level.

We considered the following hematological data at diagnosis: white blood cell (WBC), platelet (Plts), and complete blood cell counts, levels of albumin, C-reactive protein (CRP), ferritin, fibrinogen, hemoglobin (Hb), total protein, and immunoglobulins (IgG, IgA, and IgM). Tumor relapses were always confirmed by the pathologist. All blood samples (5 mL) were collected in sodium citrate vials, and centrifuged at 820× *g* for 10 min. Plasma was stored at −80 °C. An aliquot of 500 µL from each plasma sample was used for the proteomic analysis.

### 4.3. Study Design

Patients were divided into four groups: (i) an exploratory group of relapsed HL (*n* = 7); (ii) an exploratory group of non-relapsed HL (*n* = 14); (iii) a validation group of relapsed HL (*n* = 7); and (iv) a validation group of non-relapsed HL (*n* = 14). Our exploratory and validation cohorts consisted of the two exploratory and two validation groups, respectively. Label-free LC-MS/MS was used to analyze the two groups in the exploratory cohort in Part I of the study, and the two groups in the validation cohort in Part II. The abundance of selected proteins was found to differ between the two cohorts. After additional testing (i.e., immunoblotting, ELISA and Luminex-based assays) on the latter validation cohort, in Part III of the study we further examined two candidate proteins found related to innate immunity or predictive of relapse.

### 4.4. Protein Extraction and Digestion for LC-MS/MS

Proteins were extracted from 200 μL of plasma. The ProteoMiner enrichment Kit (Bio-Rad Laboratories, Hercules, CA, USA) was used to deplete the most abundant plasma proteins. The Proteominer equalization method is based on a large highly diverse combinatorial library of immobilized hexapeptides (millions of different ligands) which is mixed with the plasma samples. High abundant proteins quickly saturate their binding sites and are thus captured in limited amounts (they are washed out during the protocol), while the low abundance ones are captured and concentrated [51]. Protein concentrations were measured with the BCA assay (Thermo Fisher Scientific, Waltham, MA, USA). For the exploratory phase, a protein pool was created from all the extracted proteins for each group (relapsed and non-relapsed). For the validation phases, protein extracts from the two validation groups (relapsed and non-relapsed) were analyzed separately.

Proteins were digested in S-Trap spin columns (Protifi, Farmingdale, NY, USA) according to the manufacturer’s protocol. Briefly, around 45 μg of protein was mixed with 3% sodium dodecyl sulfate and 20 mM dithiothreitol, then boiled and alkylated with 40 mM iodoacetamide. The proteins were acidified by adding phosphoric acid (1.2% *v*/*v*), mixed with six volumes of binding buffer (100 mM ammonium bicarbonate in 90% methanol), loaded onto S-Traps, and spun at 2000 rpm. The flow-through was collected and reloaded onto the S-Trap three times. Finally, proteins were digested in trypsin (4.5 µg per sample) for 1 h at 47 °C. Hydrophilic peptides were eluted with 50 mM ammonium bicarbonate and 0.2% (*v*/*v*) aqueous formic acid, and hydrophobic peptides with 50% acetonitrile and 0.2% (*v*/*v*) formic acid. The two peptide solutions were combined for each sample, lyophilized and resuspended in 45 µL of 0.2% formic acid. All reagents were from Sigma-Aldrich (St-Louis, MO, USA).

### 4.5. LC-MS/MS and Label-Free Proteomic Profiling

The peptide mixtures were analyzed with LC-MS/MS at the Proteomics Facility of CEINGE-Biotecnologie Avanzate (Naples, Italy), using a linear ion trap LTQ Orbitrap XL mass spectrometer equipped with ETD nanoLC-MS/MS LIT-FITR (Thermo Fisher Scientific), as described previously [14] with minor modifications. Pooled samples were analyzed in triplicate for the exploratory phase, while individual samples were analyzed in duplicate for the validation phase. The tryptic peptides were loaded, concentrated, and desalted on a C18 precolumn (Thermo Scientific SC001). Each peptide sample was then fractionated in a C18 reverse-phase capillary column (Nano Separation, Niewkoop, The Netherlands) at a flow rate of 250 nL/min on a gradient from 5% to 95% buffer B (eluent B: 0.2% formic acid in 95% acetonitrile; eluent A: 0.2% formic acid and 2% acetonitrile in ultrapure water) over a period of 285 min. The mass spectrometer was run in positive polarity mode with a capillary temperature 275 °C. The LC-MS/MS analyses were performed in data-dependent acquisition (DDA) mode: a given MS scan (mass range from 400 to 1800 *m*/*z*) was followed by MS/MS scans of the 10 ions found most abundant in the previous scan, applying a dynamic exclusion window of 40 s. Relative protein amount across our samples (relapsed and non-relapsed HL) was identified and quantified through a label free quantitation. The semiquantitative LFQ approach, based on precursor ion signal intensity, was adopted after checking the good quality of precursor ion peaks (MS1) (both high abundance and low abundance signals) extracted from the full scan chromatograms. Label-free quantification (LFQ) and database searching was carried out with MaxQuant LFQ software (version 1.6.17.0, Max-Planck Institute for Biochemistry, Martinsried, Germany; www.maxquant.org; [52]) integrated with the Andromeda search algorithm and the FASTA File for *Homo sapiens* database downloaded from Uniprot (updated to January 2021). The MaxQuant software generates a LFQ intensity value, according to algorithms for normalized intensity profiles described in [53]).

Cysteine carbamidomethylation was considered as a fixed modification, and methionine oxidation as a variable modification. The mass tolerance was set to 10 ppm for precursors, and to 0.05 Da for fragment ions. Unique and razor peptides were both used for protein quantification. The false discovery rate (FDR) was set to 0.01.

Statistically significant proteins were examined using Perseus software (version 1.6.15.0, Max-Planck Institute for Biochemistry, Martinsried, Germany; https://maxquant.net/perseus/ [54]), adopting an unpaired *t*-test with a *p*-value threshold of 0.05. All potential contaminants, reverse hits, and hits found only by site were filtered out before log2 transformation of the LFQ intensities. The fold change (FC) of statistically significant proteins was calculated as the ratio of the average LFQ intensities between the relapsed and non-relapsed HL groups. Proteins differing in abundance between the two groups were defined as those with a log_2_(FC) >0.26 (proteins upregulated in relapsed patients) or ≤−0.32 (proteins downregulated in relapsed patients) in both the exploratory and the validation phases. Differences in LFQ intensities between the relapsed or non-relapsed patients subgrouped according to whether they had an adequate response (AR) or an inadequate response (IR) to treatment were tested for significance using one-way analysis of variance (ANOVA). A *p*-value < 0.05 was considered significant.

### 4.6. Protein Functional Annotation

The functional annotation of the proteins differing in abundance between the relapsed and non-relapsed pediatric/adolescent HL groups (*p* < 0.05) was carried out with DAVID 6.8 [55] for both cohorts. Gene Ontology (GO) biological processes associated with the proteins were considered together with modified *p*-values (Fisher’s exact test), and the strongly enriched annotation categories (*p* < 0.01) were considered.

### 4.7. Validation of Candidate Biomarkers

Immunoblotting was used to examine the levels of C4b-binding protein α chain (C4BPA) and clusterin (CLU)—found more abundant in relapsed HL, in both the exploratory and the validation analyses—and of α-1-antitrypsin (SERPINA1)—found less abundant in relapsed HL, and related to immunity in previous studies [14,21]. The plasma protein pools from relapsed and non-relapsed patients in the validation cohort were analyzed. Protein (15 µg per sample) was fractionated on 12% Criterion TGX Stain-Free gels (Bio-Rad), and electrotransferred onto nitrocellulose membranes after gel image acquisition with the Chemidoc system (Bio-Rad). The primary antibodies used were anti-C4BPA (1:5000; #ab200345, AbCam, Cambridge, UK), anti-CLU (1:1000; #ab69644, AbCam), and anti-α-1-antitrypsin [EPR10832(B)] (1:1000; ab167414, AbCam). The secondary was HRP-conjugated antibody (1:10,000 dilution; Bethyl). Antibody-labeled proteins were visualized using a Chemidoc imaging system and Clarity Western ECL Substrate (Bio-Rad).

Pre-therapy plasma concentrations of CLU and SERPINA1 were measured using a Luminex 200 platform including the xPONENT software (Version 3.1; Luminex Corporation, Austin, TX, USA) and two MilliPlex MAP Kits: Panel 6 #HNDG2MAG-36K for CLU, and Panel 2 #HKI6MAG-99K for SERPINA1 (EMD MilliPore, MA, USA), according to the manufacturers’ protocols. A 3000-fold dilution was optimal for CLU detection (Panel 6), while an 8000-fold dilution was needed for SERPINA1 (Panel 2). Each sample (25 μL of diluted plasma) was measured in duplicate.

## 5. Conclusions

In this case-control study, we demonstrate that pediatric/adolescent patients with HL have differences in the protein profile of their plasma at diagnosis that were associated with their likelihood of relapse after first-line treatment. Our quantitative label-free LC-MS/MS approach identified two proteins (C4BPA and CLU) in higher abundance in the plasma collected before any therapy from patients whose HL subsequently relapsed: this makes them candidate early biomarkers of the risk of relapse. These proteins are all involved in innate immune response. Our study may contribute to supporting a key role(s) for specific immune factors in the relapse of NS-HL treated under the EuroNet-PHL-C2 protocol.

## Figures and Tables

**Table 1:** Patients’ characteristics by disease status (relapsed vs. non-relapsed) after first-line treatment.

Parameter	Exploratory Cohort		Validation Cohort	
	Relapsed	Non-Relapsed	Relapsed	Non-Relapsed
Sex, *n*				
Male	5	9	3	7
Female	2	5	4	7
Age at diagnosis, years ^a^	15 (11–18)	16 (10–20)	16 (7–24)	15 (7–19)
Stage, *n*				
II	3	6	2	4
III	2	5	2	4
IV	2	3	3	6
Systemic symptoms, *n*				
Yes	4	9	2	4
No	3	5	5	10
EBV-positive, *n*	2	4	2	4
Treatment level,				
TL-2	3	7	1	3
TL-3	4	7	6	11
Response category at ERA ^b^				
AR	2	9	4	10
IR	5 *	5	3	4

^a^ mean (range); ^b^ early response assessment (ERA): AR, adequate response; IR, inadequate response; n.a., not available; * one patient had progression at ERA.

**Table 2:** Differences in the abundance of proteins in the plasma of patients by HL status (relapsed vs. non-relapsed) in the exploratory cohort (*p* < 0.05).

UniProtKB ID	Gene	Protein	-LOG (*p*-Value)	Predicted Location *	Cancer/Disease-Related Gene	Tissue Protein Expression	log_2_(FC)
**More abundant in relapsed HL (*n* = 24)**							
P04264	KRT1	Keratin, type II cytoskeletal 1	3.94	I	disease	✓	1.59
P35908	KRT2	Keratin, type II cytoskeletal 2 epidermal	3.47	I	disease	-	1.58
P01008	SERPINC1	Antithrombin-III	3.41	S	disease	✓	1.49
B9A064	IGLL5	Immunoglobulin λ-like polypeptide 5	1.91	I, S	-	pending	1.49
P01871	IGHM	Immunoglobulin heavy constant mu	3.28	I, M, S	disease	✓	0.88
Q08380	LGALS3BP	Galectin-3-binding protein	2.10	S	cancer	✓	0.88
P01591	JCHAIN	Immunoglobulin J chain	3.41	S	-	✓	0.85
P63261	ACTG1	Actin, cytoplasmic 2	2.88	I	disease	✓	0.85
P02652	APOA2	Apolipoprotein A-II	1.18	I, S	cancer	-	0.85
Q9BXR6	CFHR5	Complement factor H-related protein 5	1.51	S	disease	✓	0.84
P09871	C1S	Complement C1s subcomponent	3.73	I, S	disease	-	0.76
P02679	FGG	Fibrinogen γ chain °	1.28	I, S	cancer	✓	0.64
P01876	IGHA1	Immunoglobulin heavy constant α 1	1.18	I, M, S	disease	✓	0.62
P10909	CLU	Clusterin °	1.79	I, S	cancer	✓	0.59
P02788	LTF	Lactotransferrin	1.69	I, S	cancer	✓	0.57
P04004	VTN	Vitronectin °	2.27	S	cancer	✓	0.54
P02649	APOE	Apolipoprotein E °	1.34	S	cancer	✓	0.51
P02741	CRP	C-reactive protein	3.63	I, S	cancer	✓	0.45
O14791	APOL1	Apolipoprotein L1	1.12	M, S	disease	✓	0.39
P00450	CP	Ceruloplasmin	1.66	I, S	cancer	✓	0.39
P00734	F2	Prothrombin °	2.80	I, S	cancer	✓	0.34
P02671	FGA	Fibrinogen α chain	1.22	S	cancer	-	0.33
Q9HDC9	APMAP	Adipocyte plasma membrane-associated protein	1.19	I, M	-	✓	0.32
P04003	C4BPA	C4b-binding protein α chain °	1.31	S	cancer	✓	0.28
**More abundant in non-relapsed HL (*n* = 22)**							
P02647	APOA1	Apolipoprotein A-I	1.69	I, S	cancer	✓	−0.34
P12259	F5	Coagulation factor V	1.69	S	disease	pending	−0.42
P27169	PON1	Serum paraoxonase/arylesterase 1	1.32	S	cancer	✓	−0.45
P0C0L5	C4B	Complement C4-B	2.82	I, S	disease	✓	−0.47
P08603	CFH	Complement factor H	2.31	S	cancer/disease	✓	−0.48
P01624	IGKV3-15	Immunoglobulin kappa variable 3–15	2.55	S	-	✓	−0.49
P19823	ITIH2	Inter-α-trypsin inhibitor heavy chain H2	1.63	S	-	✓	−0.50
P07996	THBS1	Thrombospondin-1	2.36	S	cancer	✓	−0.50
P01619	IGKV3-20	Immunoglobulin kappa variable 3–20	1.39	S	-	-	−0.52
P0DJI9	SAA2	Serum amyloid A-2 protein	1.22	S	disease	pending	−0.54
P01717	IGLV3-25	Immunoglobulin λ variable 3–25	1.76	S	-	✓	−0.63
P00747	PLG	Plasminogen	2.00	S	cancer/disease	✓	−0.66
P01857	IGHG1	Immunoglobulin heavy constant γ 1	1.74	I, M, S	disease	✓	−0.68
P06727	APOA4	Apolipoprotein A-IV	1.87	S	-	✓	−0.74
P02746	C1QB	Complement C1q subcomponent subunit B	1.97	S	disease	-	−0.85
P02787	TF	Serotransferrin	3.42	I, S	cancer/disease	✓	−0.89
P01859	IGHG2	Immunoglobulin heavy constant γ 2	2.01	I, M, S	-	✓	−0.96
P01009	SERPINA1	α-1-antitrypsin	3.93	I, S	cancer	✓	−1.03
P00738	HP	Haptoglobin	2.76	I, S	cancer	✓	−1.12
P02765	AHSG	α-2-HS-glycoprotein	2.54	S	cancer/disease	✓	−1.23
P01023	A2M	α-2-macroglobulin	2.89	S	cancer	✓	−1.38
P01700	IGLV1-47	Immunoglobulin λ variable 1–47	2.83	S	-	pending	−1.42

**°** Difference in protein abundance confirmed in the validation cohort (Table S2); FC: fold change (LFQ intensity ratio between relapsed and non-relapsed HL). * generated after searching the Human Protein Atlas (www.proteinatlas.org; accessed on 2 February 2022): I, intracellular; M, membrane; S, secreted.

## Supplementary Materials

The following supporting information can be downloaded at: https://www.mdpi.com/article/10.3390/ijms23179911/s1.

## Abbreviations

AR	adequate response to therapy on early response assessment
C4BPA	C4b-binding protein α chain
CLU	clusterin
COPDAC-28	consolidation chemotherapy cycle: cyclophosphamide, vincristine, prednisone and dacarbazine, given every 28 days
COPP	chemotherapy cycle: cyclophosphamide, vincristine, prednisone and procarbazine
DECOPDAC-21	consolidation chemotherapy cycle: cyclophosphamide, vincristine, prednisone, etoposide, doxorubicin and dacarbazine, given every 21 days
FDR	false discovery rate
ELISA	enzyme-linked immunosorbent assays
ERA	early response assessment
GO	Gene Ontology
HL	Hodgkin lymphoma
IR	inadequate response to therapy on early response assessment
LC-MS/MS	liquid chromatography coupled to tandem mass spectrometry
LFQ	label-free quantification
NR	non-relapsed
OEPA	induction chemotherapy cycle: vincristine, etoposide, prednisone and doxorubicin, given every 28 days
R	relapsed
SERPINA1	α-1-antitrypsin
