# Supplementary material for: Quantitative Plasma Proteomics to Identify Candidate Biomarkers of Relapse in Pediatric/Adolescent Hodgkin Lymphoma
Source: Int J Mol Sci. 2022 Aug 31;23(17):9911. doi: 10.3390/ijms23179911 (PMC9456176; doi:10.3390/ijms23179911)
Supplement: Supplementary file 1 [file ijms-23-09911-s001.zip › Supplementary Tables.pdf]

Table S1. Hematological characteristics of patients with pediatric/adolescent Hodgkin lymphoma relapsed or non-relapsed.

Parameter	Explorative cohort			Validation cohort		
	Relapsed	Non-relapsed	*p-value	Relapsed	Non-relapsed	*p-value
WBC (10^3/μL)	17.13 ± 9.01	10.75 ± 4.70	0.056	11.93 ± 4.74	10.17 ± 5.21	0.36
Lymphocytes (10^3/μL)	1.78 ± 0.45	1.27 ± 0.58	0.091	1.02 ± 0.45	2.16 ± 3.19	0.31
Plts 10^9/L	405.60 ± 140.64	317.70± 135.65	0.173	383.00 ± 107.38	352.33 ± 148.74	0.51
Albumin (g/dL)	3.58 ± 0.67	4.04 ± 0.32	0.795	3.30 ± 0.39	3.69 ± 0.58	0.60
C-reactive protein (mg/dL)	4.97 ± 5.34	3.30 ± 2.270	0.330	8.67 ± 7.85	9.03 ± 6.19	0.95
Ferritin (ng/mL)	283.00 ± 283.34	154.59 ± 59.26	0.168	309.83 ± 124.11	298.08 ± 266.19	0.75
Fibrinogen (mg/dL)	435.50 ± 101.88	490.09 ± 93.52	0.755	559.67 ± 173.39	565.29 ± 203.83	0.86
Hb (g/dL)	10.88 ± 1.72	12.50 ± 1.57	0.664	10.64 ± 2.31	10.49 ± 1.64	0.55
Total protein (g/dL)	7.40 ± 0.70	7.97 ± 0.66	0.972	7.62 ± 0.69	7.64 ± 1.15	0.60
IgA (mg/dL)	210.50 ± 137.43	212.55 ± 89.62	0.899	246.00 ± 147.41	239.58 ± 121.80	0.80
IgG (mg/dL)	1572.25 ± 482.69	1603.09 ± 313.66	0.800	1238.71 ± 411.11	1426.85 ± 547.16	0.59
IgM (mg/dL)	146.00 ± 37.59	115.82 ± 52.50	0.227	95.43 ± 46.66	124.31 ± 75.05	0.40

Ptls: platelet count; Hb: hemoglobin; IgG: immunoglobulin. Data are presented as mean ± standard deviation of the mean. * p-value > 0.05 for all.

Table S2. Proteins differing in their abundance in plasma from patients with relapsed vs non-relapsed pediatric/adolescent HL, in the validation cohort (p <0.05)

UniProtKB ID	Gene	Protein	-LOG(p-value)	Predicted location *, °°	Cancer/disease related gene *, †	Tissue protein expression *	log_2(FC)
**More abundant in non-relapsed HL (n =23)**							
P02753	RBP4	Retinol-binding protein 4	3.91	I, S	cancer/disease	✓	2.01
P55056	APOC4	Apolipoprotein C-IV	4.79	S	-	-	1.34
P02751	FN1	Fibronectin	2.43	I, S	cancer/disease	✓	1.24
P22792	CPN2	Carboxypeptidase N subunit 2	5.58	S	-	✓	1.14
P02760	AMBP	Protein AMBP	2.77	I, S	-	✓	0.90
Q9UK55	SERPINA 10	Protein Z-dependent protease inhibitor	3.52	M, S	-	pending	0.89
P27918	CFP	Properdin	2.99	I, S	disease	pending	0.75
P00742	F10	Coagulation factor X	2.72	S	disease	pending	0.67
P04070	PROC	Vitamin K-dependent protein C	3.61	I, S	cancer/disease	✓	0.67
P02766	TTR	Transthyretin	3.30	I, S	cancer/disease	✓	0.66
P00734	F2	Prothrombin°	5.68	I, S	cancer	✓	0.59
P03952	KLKB1	Plasma kallikrein	2.33	I, S	disease	pending	0.53
P22352	GPX3	Glutathione peroxidase 3	2.37	I, S	-	✓	0.53
P02743	APCS	Serum amyloid P-component	2.78	S	disease	✓	0.52
P05090	APOD	Apolipoprotein D	2.21	S	cancer	✓	0.51
P12259	FA5	Coagulation factor V	2.21	S	disease	✓	0.50
O14791	APOL1	Apolipoprotein L1	2.29	M, S	cancer/disease	cancer/disease	0.49
P20851	C4BPB	C4b-binding protein beta chain	4.87	S	disease	✓	0.48
P04180	LCAT	Phosphatidylcholine-sterol acyltransferase	2.49	I, S	disease	pending	0.46
P02679	FGG	Fibrinogen gamma chain°	2.44	I, S	cancer/disease	✓	0.45
P10909	CLU	Clusterin°	3.19	I, S	cancer	✓	0.44
P04003	C4BPA	C4b-binding protein alpha chain°	2.68	S	cancer	✓	0.37
P04004	VTN	Vitronectin°	2.89	S	cancer	✓	0.33

Proteins also differing in abundance in the exploratory cohort (Table 2); FC: fold change (ratio in LFQ intensity values between relapsed and non-relapsed HL). *after searching the Human Protein Atlas (www.proteinatlas.org; 02-Feb-2022). °° I, intracellular; M, membrane; S, secreted. † Protein class: cancer- or disease-related gene.

Table S3. Protein levels of α-1-antitrysin and clusterin measured using the Luminex system in plasma of pediatric/adolescent patients with relapsed and non-relapsed HL, in the validation cohort.

Analyte	Relapsed (*n* = 7)	Non-Relapsed (*n* = 14)	*p*-value
Alpha-1-antitrypsin (mg/dL)	58.117 ± 14.179	378.356 ± 703.578	0.270
Clusterin (μg/mL)	914.435 ± 312.749	657.992 ± 138.800	0.016

Data are presented as means ± standard deviations of the mean.
