# Supplementary figures and images for: Quantitative Plasma Proteomics to Identify Candidate Biomarkers of Relapse in Pediatric/Adolescent Hodgkin Lymphoma
Source: Int J Mol Sci. 2022 Aug 31;23(17):9911. doi: 10.3390/ijms23179911 (PMC9456176; doi:10.3390/ijms23179911)

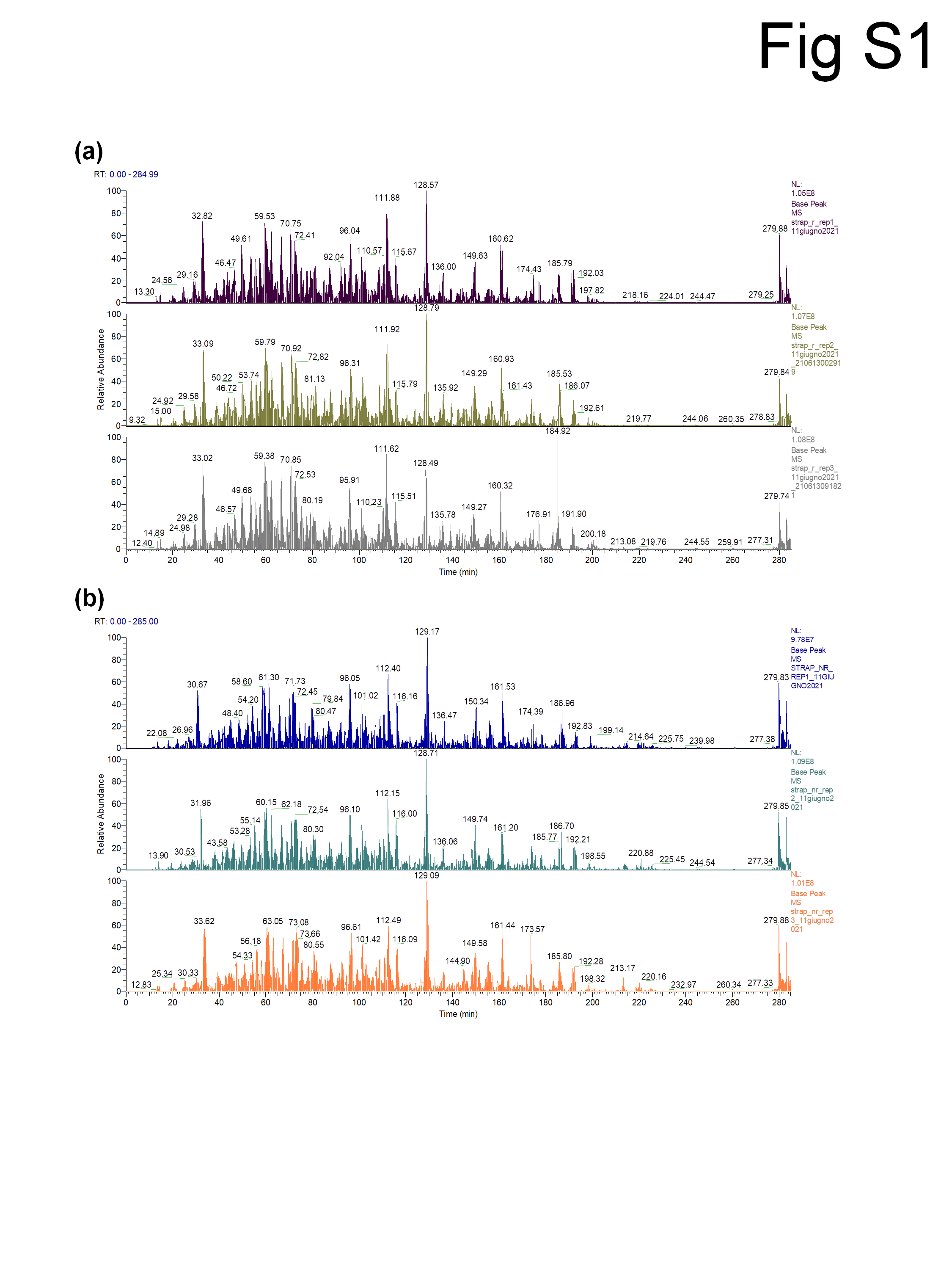

Supplement: Supplementary file 1 [file ijms-23-09911-s001.zip › Fig S1.tif]

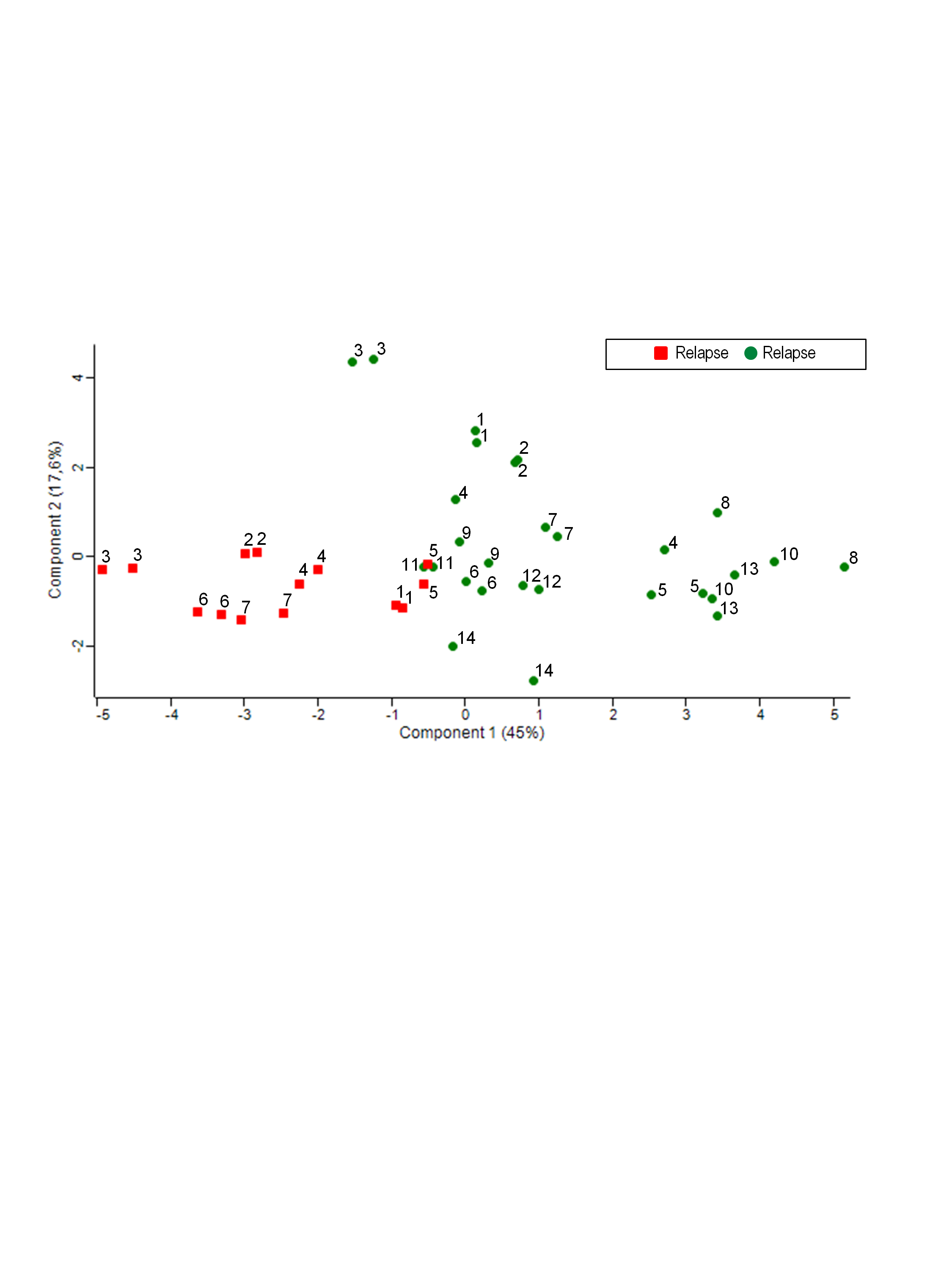

Supplement: Supplementary file 1 [file ijms-23-09911-s001.zip › Fig S2.tif]

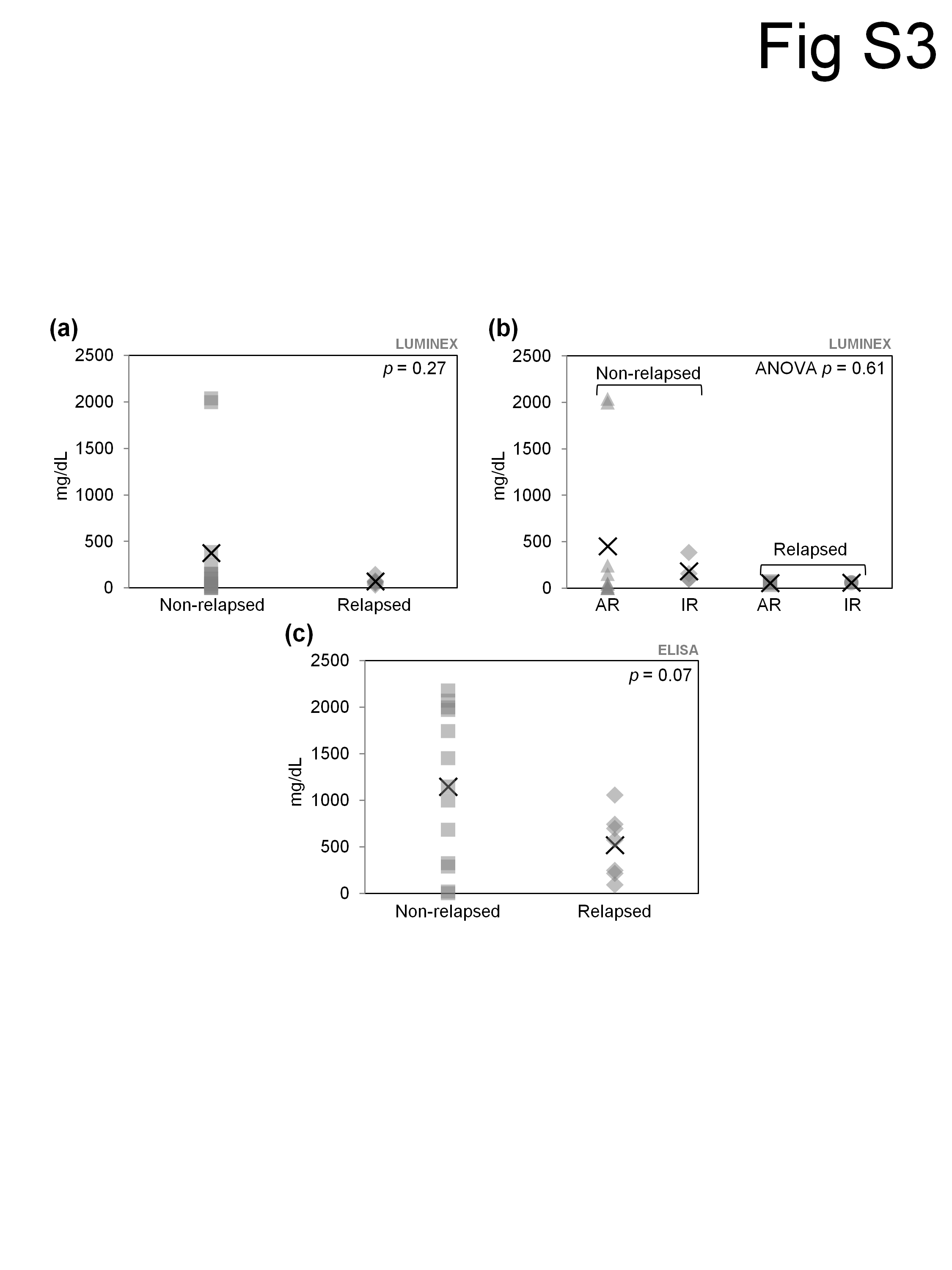

Supplement: Supplementary file 1 [file ijms-23-09911-s001.zip › Fig S3.tif]
